# Sternal allograft transplantation for anterior chest wall reconstruction

**DOI:** 10.1186/1749-8090-8-S1-O322

**Published:** 2013-09-11

**Authors:** N Asadi, A Dell'Amore, G Dolci, D Greco, G Caroli, C Ammari, A Bini, F Stella

**Affiliations:** 1 Thoracic Surgery, S. Orsola-Malpighi Hospital, University of Bologna, Italy

## Background

Reconstruction of the chest wall after an anterior chest wall resection remains a difficult and controversial issue, therefore a correct reconstruction of these large resections is fundamental to avoid secondary complications. Different materials have been used to reconstruct the sternum, but none of them are considered the gold standard procedure. We propose a new technique using sternal-allograft to reconstruct the anterior chest wall after sternal resection.

## Methods

The sternal allograft was harvested from a multi-tissue donor. After packaging, the allograft was stored at -80°C until it’s use. After a complete or partial resection of the sternum en-bloc with costal cartilage the sternal allograft was tailored to perfectly fit with chest wall defect and then the allograft was fixed using titanium bars and screws.

## Results

Between June 2010 and February 2013, seven patients underwent sternectomy followed by anterior chest-wall reconstruction using sternal transplantation. Six patients were treated for neoplastic diseases, one patient for complete sternal dehiscence after sternotomy for cardiac surgery. In the post-operative period no major complications were reported and the patients were discharged after 9±2,4 days. The mean follow-up time was 23.6±4.1 months. No mechanical failure or reconstruction related complications have been reported during follow-up and all patients are alive, in good clinical conditions, pain free and with normal pulmonary function tests.

## Conclusions

The sternal allograft transplantation technique is simple and useful technique providing excellent functional and cosmetic results without complications during the follow-up.

